# Prevalence and predictors of hospital prealerting in acute stroke: a mixed methods study

**DOI:** 10.1136/emermed-2014-204392

**Published:** 2016-02-23

**Authors:** J P Sheppard, A Lindenmeyer, R M Mellor, S Greenfield, J Mant, T Quinn, A Rosser, D Sandler, D Sims, M Ward, R J McManus

**Affiliations:** 1 Nuffield Department of Primary Care Health Sciences, NIHR School for Primary Care Research, University of Oxford, Oxford, Oxfordshire, UK; 2 Primary Care Clinical Sciences, NIHR School for Primary Care Research, University of Birmingham, Birmingham, West Midlands, UK; 3 Department of Public Health, NHS Lanarkshire, Bothwell, UK; 4 Primary Care Unit, University of Cambridge, Cambridge, Cambridgeshire, UK; 5 Faculty of Health, Social Care and Education, St George's, University of London & Kingston University, London, UK; 6 West Midlands Ambulance Service NHS Trust, Regional Ambulance Headquarters, Dudley, West Midlands, UK; 7 Heart of England NHS Foundation Trust, Birmingham, West Midlands, UK; 8 Queen Elizabeth Hospital Birmingham Elderly Care, University Hospitals Birmingham NHS Foundation Trust, Birmingham, West Midlands, UK

**Keywords:** prehospital care, emergency department

## Abstract

**Background:**

Thrombolysis can significantly reduce the burden of stroke but the time window for safe and effective treatment is short. In patients travelling to hospital via ambulance, the sending of a ‘prealert’ message can significantly improve the timeliness of treatment.

**Objective:**

Examine the prevalence of hospital prealerting, the extent to which prealert protocols are followed and what factors influence emergency medical services (EMS) staff's decision to send a prealert.

**Methods:**

Cohort study of patients admitted to two acute stroke units in West Midlands (UK) hospitals using linked data from hospital and EMS records. A logistic regression model examined the association between prealert eligibility and whether a prealert message was sent. In semistructured interviews, EMS staff were asked about their experiences of patients with suspected stroke.

**Results:**

Of the 539 patients eligible for this study, 271 (51%) were recruited. Of these, only 79 (29%) were eligible for prealerting according to criteria set out in local protocols but 143 (53%) were prealerted. Increasing number of Face, Arm, Speech Test symptoms (1 symptom, OR 6.14, 95% CI 2.06 to 18.30, p=0.001; 2 symptoms, OR 31.36, 95% CI 9.91 to 99.24, p<0.001; 3 symptoms, OR 75.84, 95% CI 24.68 to 233.03, p<0.001) and EMS contact within 5 h of symptom onset (OR 2.99, 95% CI 1.37 to 6.50 p=0.006) were key predictors of prealerting but eligibility for prealert as a whole was not (OR 1.92, 95% CI 0.85 to 4.34 p=0.12). In qualitative interviews, EMS staff displayed varying understanding of prealert protocols and described frustration when their interpretation of the prealert criteria was not shared by ED staff.

**Conclusions:**

Up to half of the patients presenting with suspected stroke in this study were prealerted by EMS staff, regardless of eligibility, resulting in disagreements with ED staff during handover. Aligning the expectations of EMS and ED staff, perhaps through simplified prealert protocols, could be considered to facilitate more appropriate use of hospital prealerting in acute stroke.

**Key messages**

**What is already known on this subject?**

- Thrombolysis can significantly reduce the burden of stroke, but the time window for safe and effective treatment is short. Where patients travel to hospital via ambulance, the sending of a ‘prealert’ message to the ED can significantly improve the timeliness of treatment upon arrival in hospital. The number of patients being prealerted in routine clinical practice varies widely and there is a paucity of research examining why such variation exists.

**What might this study add?**

- Up to half of the patients presenting with suspected stroke are prealerted by the emergency medical services, in some cases, against the instruction of locally agreed rapid transfer protocols, resulting in disagreements with hospital staff during handover. Better understanding and communication of prealert protocols, and the principles that underlie them, are needed to facilitate collaborative working between all staff involved in the acute stroke pathway.

## Introduction

Stroke is estimated to cause approximately 5.7 million deaths worldwide and the loss of up to 50 million disability-adjusted life years every year.^1^ Thrombolysis, using alteplase, can significantly reduce the burden of ischaemic stroke (which accounts for approximately 80% of all strokes) but the time window for safe and effective treatment is short.^2^ Thrombolysis results in improved functional outcome if it is administered within 6 h of symptom onset^3^ but currently, only around 5%–13% of patients with stroke in developed countries receive treatment.^4^ Access to thrombolysis requires timely arrival in hospital and the emergency medical services (EMS) are key to ensuring patients arrive quickly.^5^

Where patients travel to hospital via ambulance, the sending of a ‘prealert’ message to the ED informing them that a patient with potential stroke is en route has been shown to improve the timeliness of subsequent treatment upon arrival in hospital.^6^ ^7^ However, the number of patients being prealerted varies widely (22%–67%)^6–8^ and it is unclear why such variation exists. Previous studies have examined the influence of patient characteristics upon arrival in hospital (recorded in hospital records) on the prevalence of hospital prealerting and found that increasing age, ethnicity, medical history and increasing stroke severity were among the key independent predictors of prealerting.^8^ ^9^ However, to our knowledge, no previous studies have examined how the initial prehospital patient presentation affects prealerting, which may be different. There is no previous work describing the influence of localised prealert protocols on health service provider behaviour.

This study used both quantitative and qualitative methodologies to examine the prevalence of hospital prealerting in routine clinical practice, the extent to which prealert protocols are being followed and contextual factors associated with EMS staff's decision to send a prealert message in acute stroke.

## Methods

### Study design and setting

This was a retrospective cohort study using both quantitative and qualitative methodologies. It was conducted as part of a larger project for which the process of recruitment and data collection have been detailed elsewhere.^10^ The study was carried out in two urban hospitals (West Midlands, UK). Both hospitals offered an acute stroke service: 24 h a day at one site and in working hours (09:00 to 17:00, Monday to Friday) in the other. Patients from either hospital catchment were eligible if they contacted the EMS and were transported to hospital by the local ambulance service. A summary of the patient pathway for acute stroke in the UK is detailed in the online supplementary figure S1. At the time of the study, a 4.5 h maximum time window for thrombolysis was in operation.

10.1136/emermed-2014-204392.supp1 Supplementary figure 1 Patient pathway for acute stroke in UK hospitals offering a stroke service 24 hours a day, 7 days a week

### Eligibility for prealert

Patients were deemed eligible for prealert according to a localised EMS protocol which was in place to ensure the rapid transfer of patients with suspected stroke who were potentially eligible for thrombolysis (see online supplementary figure S2). This protocol required EMS staff to ensure that patients satisfied all five eligibility criteria:

1. have a positive Face, Arm, Speech Test (FAST)^11^
2. have a known symptom onset time within 5 h
3. high level of consciousness (defined here by the authors and expert collaborators as a GCS of >13)
4. blood glucose of >3 mmol/L
5. no evidence of seizure or fit

10.1136/emermed-2014-204392.supp2 Supplementary figure 2 Local hospital pre-alert criteria

The protocol was devised and agreed by members of the local ambulance and specialist stroke services and regional stroke network.

### Quantitative study

Patients with a suspected diagnosis of stroke who had been admitted to an acute stroke ward under the care of participating consultant stroke physicians were approached for consent by a member of the research team during their stay on the acute stroke ward between 1 May 2012 and 28 February 2013. Informed consent was obtained from all patients to permit identifiable patient data to be collected (to allow for data linkage) and those with capacity were approached. Where a patient lacked capacity, consent from an appropriate consultee was sought. An appropriate consultee was defined as someone who knew the participant in a personal capacity who was able to advise the researcher about the person's wishes and feelings in relation to the project and whether they should join the research.

The records of all consenting patients were reviewed by members of the research team. Identifiable patient data were used to locate and link hospital and EMS records. Data relating to patient demographics, prehospital assessments and whether a prealert message was sent were extracted from both EMS and hospital records. Descriptive statistics were used to describe the study population, the proportion of patients accessing acute stroke services via ambulance and the proportion of patients who were prealerted. Complete data were available for all but one patient in the cohort who was excluded from the regression modelling. No attempt was made to impute these missing data.

A logistic regression model was constructed to examine the association between prealert eligibility, patient and service factors and whether or not a prealert message was sent. Potential candidate predictors were identified from previous studies^8^ ^9^ and by a panel of experts (made up of patients with stroke, specialist stroke physicians and EMS staff). These included patient characteristics (age, sex and ethnicity), eligibility for prealert (paramedic arrival within 5 h, number of FAST symptoms, GCS, blood glucose, evidence of seizure and evidence of fit) and service factors (grade of paramedic in attendance, hospital site, time of patient presentation and final diagnosis). Blood glucose and evidence of seizure were subsequently excluded from the final model due to colinearity. Sensitivity analyses were conducted examining the multivariate model including all patient and service factors but with prealert eligibility entered as a single variable.

All statistical analyses were performed in STATA (V.13.1 MP64, StataCorp LP, Texas, USA). Data are presented as means or medians (SD, IQR or 95% CI), percentage of the recruited population (unless otherwise stated) and ORs (95% CI).

### Qualitative study

A total of seven EMS staff were recruited to the qualitative study. They were recruited through a sign-up sheet following a presentation at a national EMS conference (which included EMS staff from other Trusts). Of the 30 attendees, four signed up immediately, two further EMS staff were encouraged to participate by a colleague and one additional participant was known to the research team through the larger Collaborations for Leadership in Applied Health Research and Care stroke study.^10^ In qualitative research, a small sample size can be sufficient to study experience of a clearly circumscribed phenomenon (ie, prealerting for stroke).^12^ The final sample was found to contain a sufficient range of skill levels and experience to conform to that planned from the purposive sampling strategy.

Semistructured interviews with EMS staff were conducted using a topic guide by one trained female interviewer (RMM; the topic guide is available in the online supplementary material). EMS staff were asked about their experiences of seeing patients with suspected stroke, with particular emphasis on the decisions they made during the prehospital phase of the patient journey. One participant was interviewed on their own; the remaining six consisted of three pairs of colleagues on the same shift and therefore selected the option to be interviewed with their colleague. Participants interviewed together knew each other well and were of comparable seniority and so were able to speak freely. As each question was first answered by both participants separately, often followed-up with a wider discussion between the participants, we were able to capture seven unique viewpoints. Participants were interviewed once, either in their place of work or their home; all interviews were conducted between January 2011 and July 2013. Interviews were audio recorded and transcribed verbatim.

The quantitative component informed the qualitative topic guide development, encouraging further discourse around the topics of deciding on who to prealert and the handover between paramedic and hospital staff. The aim of the qualitative component was to provide insight into the paramedics’ reasoning for the quantitative findings.^13^

Transcripts were managed using NVivo (V.9, QSR International, Victoria, Australia). Coding was initially established using the ‘one sheet of paper’ method where all responses in a section of the interviews are summarised and compared with identify the range of responses.^14^ Themes were developed by a comparative process focusing on differences and similarities between sections of data.^15^ Further thematic analyses were conducted concurrent with data collection, which allowed an inductive approach, so that later interviews built on or queried knowledge gained from earlier data collection. Data collection was continued until a range of responses had been collected; however it was not deemed necessary to achieve data saturation as our analysis aimed to contextualise the quantitative results rather than develop theory.^16^

To ensure analytical rigour, both AL and RMM coded and double coded a sub-set of interviews, meeting regularly to compare findings and resolve differences through discussion. Quotations give participants’ identifier and pseudonym.

### Ethical approval

Full ethical approval for this project was obtained from the National Research Ethics Service Committee, London—Queen Square (reference; 09/H0716/71).

## Results

### Characteristics of the study participants

**Figure 1:**
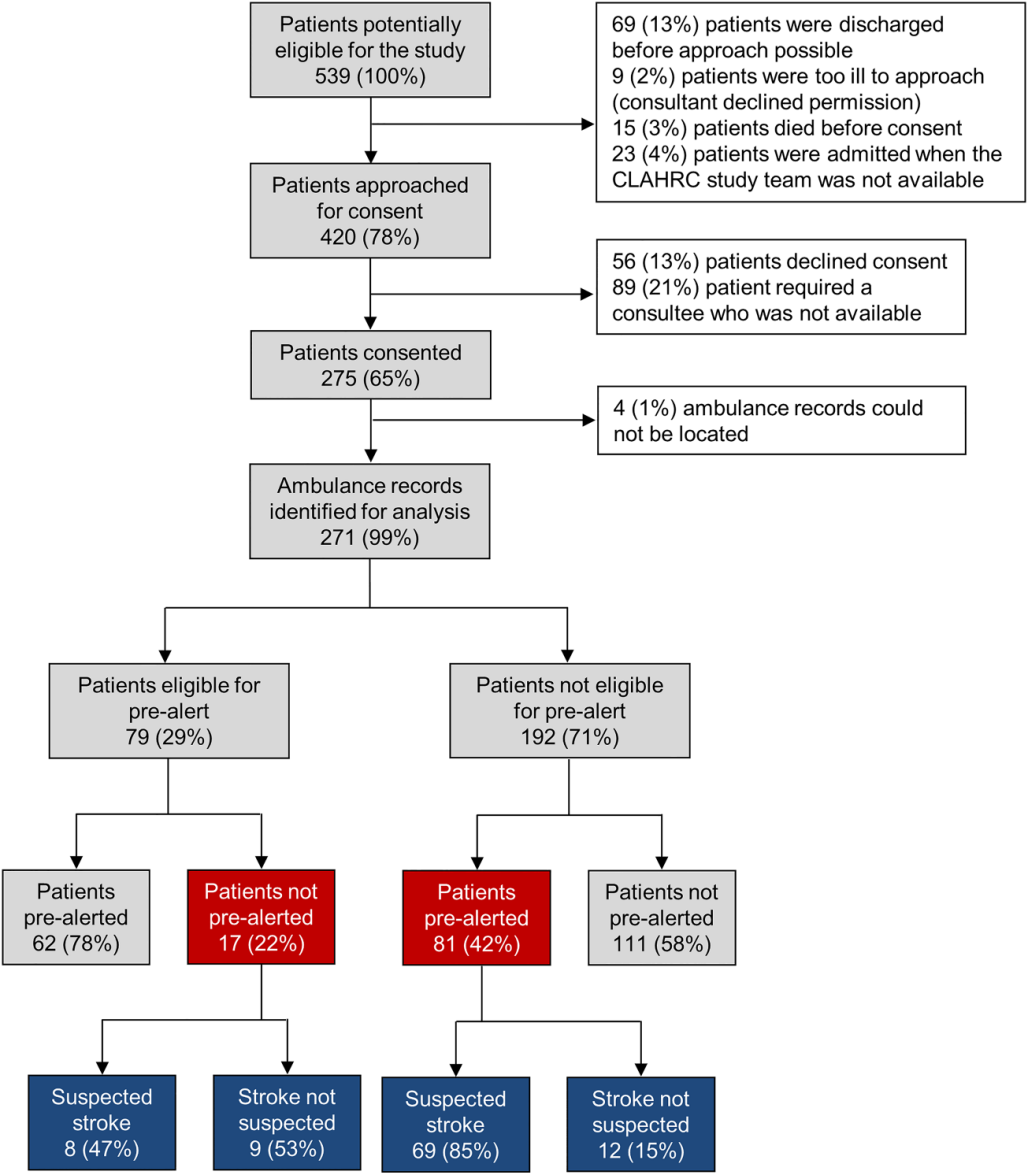
Proportion of patients who were eligible, consented, prealerted and/or were suspected of having stroke by the attending emergency medical service staff member. Eligibility for prealert defined as a FAST-positive patient, who is attended by the emergency medical services within 5 h of symptom onset, has a GCS of ≥13, a blood glucose of ≥3 mmol/L and who has not suffered a fit or a seizure. CLAHRC study, Collaborations for Leadership in Applied Health Research and Care study;^10^ FAST, Face, Arm, Speech Test.

A total of 539 patients travelling to hospital via the EMS with suspected stroke were admitted to the acute stroke wards during the recruitment period. Of these, 420 (78% of those eligible) were approached and 275 (65% of those approached) were recruited (figure 1). Patients were not approached if they were too ill (according to the judgement of the participating stroke physician), were admitted to the ward when a member of the research team was not available and were discharged or died before being approached. Four (1%) patients had to be excluded because their ambulance records could not be located, leaving 271 patient records for inclusion in the final analysis (figure 1).

Recruited patients were similar to those not recruited during the study period for all recorded demographics (table 1). Included patients were elderly (mean age 73±14 years) and the majority were male (55%) and of white ethnic origin (80%). A total of 31 patients (11%) received thrombolysis and the median time spent in hospital was 11 days (IQR, 18 days). Four of the seven EMS staff (all paramedics) interviewed for the qualitative study were men and, on average, had 10.6±7.3 years of experience in their role (table 2).

**Table 1:** Characteristics of non-recruited and recruited patients with suspected stroke

Characteristic	Non-recruited population	Recruited population
Population (n)	264 (100%)	275 (100%)
Age (mean±SD)	74±15	73±14
Gender (% female)	144 (55%)	125 (45%)
Ethnicity (%)		
White	190 (72%)	221 (80%)
South Asian	45 (17%)	36 (13%)
Black	14 (5%)	8 (3%)
Other	4 (2%)	3 (1%)
Not stated	10 (4%)	4 (1%)
Median time in hospital (inter quartile range)	Not available	11 (18)
Patients receiving thrombolysis (%)	Not available	31 (11%)
Died in hospital (%)	17 (6%)	20 (7%)

**Table 2:** Characteristics of emergency medical service staff interviewed

Category	Number of staff
Total number of staff interviewed	7
Gender	
Male	4 (57%)
Female	3 (43%)
Years of experience in the role	
0–9	4 (57%)
10–19	2 (29%)
20+	1 (14%)
Interview format	
Individual interview	1 (14%)
Two participants interviewed together	6 (86%)

### Prevalence and predictors of prealerting

Within our recruited cohort, a total of 79 (29%) patients met all five criteria and thus were eligible for prealerting (figure 1). A total of 143 (53%) patients were actually prealerted; 62 (43%) of these satisfied all five eligibility criteria (table 3). Seventeen patients were not prealerted, despite being eligible for prealert. Of those who were not eligible, 81 (42%) were prealerted.

**Table 3:** Characteristics of those patients who were and were not prealerted

Characteristic	Not prealerted	Prealerted
Population (n)	128 (100%)	143 (100%)
Age (mean±SD)	72±14	75±13
Gender (% female)	54 (42%)	74 (52%)
Ethnicity (%)		
White	102 (80%)	110 (77%)
Mixed race	2 (2%)	3 (2%)
Asian or Asian British	13 (10%)	17 (12%)
Black or Black British	4 (3%)	3 (2%)
Other	6 (5%)	9 (6%)
Not stated	1 (1%)	1 (1%)
Patients seen by the EMS within 5 h of symptom onset (%)	53 (41%)	93 (65%)
Patients who were FAST positive (%)	48 (38%)	129 (90%)
Patients with a GCS >13 (%)	119 (93%)	89 (62%)
Patients with a blood glucose >3 (%)	124 (97%)	143 (100%)
Patients who had not had a seizure (%)	128 (100%)	142 (99%)
Patients who had not had a fit (%)	127 (99%)	141 (99%)
Fulfils local criteria for prealert (%)	17 (13%)	62 (43%)
Patients who had a paramedic in attendance (%)	99 (92%)	115 (93%)
Final diagnosis (%)		
Stroke	110 (86%)	134 (94%)
Stroke mimic	8 (6%)	5 (3%)
TIA	9 (7%)	4 (3%)
TIA mimic	1 (1%)	0 (0%)
Patients arriving in hospital in working hours (09:00–17:00) (5%)	79 (62%)	88 (62%)
Patients receiving thrombolysed (%)	4 (3%)	27 (19%)
Died in hospital (%)	4 (3%)	14 (10%)

EMS, emergency medical services; FAST, Face, Arm, Speech Test; TIA, transient ischaemic attack.

In the multivariate analysis, independent predictors of prealerting were: any number of FAST symptoms (0 symptoms, reference category; 1 symptom, OR 6.14, 95% CI 2.06 to 18.30, p=0.001; 2 symptoms, OR 31.36, 95% CI 9.91 to 99.24, p<0.001; 3 symptoms, OR 75.84, 95% CI 24.68 to 233.03, p<0.001) and, being seen by the EMS within 5 h of symptom onset (OR 2.99, 95% CI 1.37 to 6.50 p=0.006) (table 4). A GCS of >13 was inversely related to the likelihood of being prealerted (OR 0.04, 95% CI 0.01 to 0.14, p<0.001). Compared with non-prealerted patients, a higher proportion of prealerted patients were FAST positive (table 3). Entered into a separate multivariate model as a single predictor (without adjusting for individual eligibility criteria), eligibility for prealerting was not a significant independent predictor of prealerting (OR 1.92, 95% CI 0.85 to 4.34, p=0.12).

**Table 4:** Multivariate logistic regression examining factors associated with hospital prealerting in acute stroke

	Multivariate analysis		
Predictor	OR	95% CIs	p Value
*Patient characteristics*			
Age	1.00	0.97 to 1.03	0.99
Sex (male)	0.49	0.21 to 1.13	0.10
White ethnicity (reference category)	1.00	–	–
Black or Black British ethnicity	3.10	0.30 to 31.62	0.34
Asian or Asian British ethnicity	0.79	0.24 to 2.65	0.71
Mixed ethnicity	1.17	0.11 to 12.41	0.90
Other ethnicity	1.68	0.27 to 10.66	0.58
Ethnicity not stated	2.01	0.09 to 43.04	0.66
Eligibility for prealert*			
Paramedic arrives within 5 h (yes)	2.99	1.37 to 6.50	0.006
No FAST symptoms present (reference category)	1.00	–	–
1 FAST symptom present	6.14	2.06 to 18.30	0.001
2 FAST symptoms present	31.36	9.91 to 99.24	<0.001
3 FAST symptoms present	75.84	24.68 to 233.03	<0.001
GCS >13	0.04	0.01 to 0.14	<0.001
Evidence of fit (yes)	0.23	0.01 to 6.40	0.39
*Service factors*			
Highest grade of EMS staff in attendance (paramedic)	1.35	0.81 to 2.25	0.25
Hospital site (1 of 2)	2.22	1.00 to 4.90	0.05
Hospital arrival within working hours (09:00–17:00) (yes)	0.73	0.34 to 1.57	0.43
Stroke final diagnosis (stroke)	2.09	0.56 to 7.78	0.27

One patient was excluded from this analysis due to missing data relating to age.

Being FAST positive was a significant predictor of prealert using the likelihood ratio test (p<0.001), but ethnicity was not (p=0.91).

*Blood glucose and evidence of seizure variables were removed from the model due to colinearity.

EMS, emergency medical services; FAST, Face, Arm, Speech Test.

### Qualitative results

All seven paramedics interviewed for the qualitative study mentioned ‘FAST positive’ when asked when they would prealert the hospital. However, further discussion clarified that the interviewees often saw prealerting as signifying the seriousness of the patients’ condition and the need to treat them quickly. Three subthemes linked to this overarching topic were identified: (1) FAST symptoms were not always straightforward and paramedics reported that they might err on the side of caution; (2) while EMS staff reported that they used prealert to signal urgency, how the ED responded was out of their control and often a source of frustration and (3) this frustration could be exacerbated when the patient did not conform to the time-to-thrombolysis criteria.

#### The presence of ‘FAST’ symptoms

EMS staff described patients that clearly had a stroke as they were FAST positive with multiple or severe symptoms:

FAST test positive, proper FAST test positive, real, real, slurred speech, real rabbit eyes in the head, like fear all over his face (Paramedic 2, Nora)

I call them ‘aura filled’ patients, because they're not quite sure where they are … unable to speak (Paramedic 4, Jack)

However, this was not always the case and the EMS staff we interviewed reported that many patients were more difficult to recognise, especially if the patient had less pronounced symptoms or symptoms that were wearing off (possible transient ischaemic attack). EMS staff suggested they would prealert the hospital anyway but some felt that while ‘a good team will trust your judgment’, hospitals did not encourage prealerts when they were not completely sure but suspecting a stroke. Some were also aware that not every patient with stroke is FAST positive and that sometimes they had to prealert even if unsure, although this was accepted more readily for cardiac patients than stroke patients:

…with PCIs [Percutaneous Coronary Interventions] … they will always say, even if you're unsure or you're not entirely sure just bring them, alert us anyway (Paramedic 5, Ken)

#### Concerns about ED response to prealerting

As the prealert signalled the urgency of the case, a lack response of the ED to a prealert message raised concerns among the EMS staff we interviewed:

[ED staff would ask] “What have you got?”

[I would respond] “FAST positive. FAST positive alerted. And then that should be that little bit more …[but]… it does depend who you get as to whether they even look at you.” (Paramedic 2, Nora)

EMS staff reported that while it was down to the ED staff's clinical judgement how they responded to the prealert, the service could be improved by:

being able to directly contact the stroke co-ordinator where available or “*cut out the middle man”* (Paramedic 5, Ken)

or by formalising the Emergency Department response as

they are not audited on the speed of their response to the pre-alert, while paramedics and stroke units are judged harshly on their accuracy and speed in dealing with stroke patients. (Paramedic 4, Jack)

#### Misunderstandings and disagreements about prealert criteria

The quantitative findings show that EMS staff often prealerted for patients who were not eligible according to prespecified criteria. These qualitative data suggest one possible reason could be that patients were prealerted even when symptom onset time was unknown to signify the seriousness of the case, because the consequences of delaying access to specialist care could be devastating:

I would [pre-alert]. Even if -- you know, no onset, no witness, this is an unwitnessed event, found by a carer … even if they are outside the criteria I will let the hospital know because you've only got one brain. (Paramedic 1, Nina)

If you find somebody's had a stroke and you think it could probably be 12 hours, you still alert them because they're still FAST test positive but as far as the hospital's treatments are concerned, they're not going to do anything because of the time frames, out the window.

(Paramedic 6, Kylie)

However, some of the EMS staff we interviewed, while suggesting they would prealert FAST-positive patients outside of the thrombolysis time window, decided on whether to aim for maximum speed depending on the time of symptom onset:

If I've got an onset time I will blue light them in. If I know they're out of the window and they're stable but still with symptoms, we're not going to hang about, but [not going for] the two wheels round the corner run [into the ED]. (Paramedic 2, Nora)

As outlined above, EMS staff reported that they had prealerted a patient as FAST positive to ensure prompt handover of a patient they considered as serious, but the ED response could slow things down, especially if the ED was busy. A lack of consensus on the length of the thrombolysis time window could lead to frustration for EMS staff:

[The time window] is apparently three hours if they're over 80. So as he'd already been sort of two, two and a half hours, by the time we got him there … we've alerted in and everybody is there waiting for us…the bloke was definitely FAST positive, there was no question about that. And while I'm booking him in, it's oh look, he's 80 years and 11 months. Huge sigh of relief, we can slow down a bit. (Paramedic 4, Jack)

These qualitative findings reflect the complexity of a medical field where symptoms can be ambiguous and the consequences of decisions made by EMS staff can be huge. Overall, we found a disjoint between the original rationale behind the prealert protocol which was to enable timely thrombolysis, and the way it was used by the EMS staff we interviewed to signal that their patient needed to be treated with urgency.

## Discussion

### Main findings

The present study examined linked medical records from recruited patients presenting in hospital with suspected stroke and found that up to half are prealerted by EMS staff, in some cases, against the instruction of locally agreed rapid transfer protocols. Both quantitative and qualitative investigations revealed that EMS staff are more likely to prealert patients with suspected stroke with multiple FAST symptoms, irrespective of other prealert eligibility criteria. Indeed, EMS staff's interpretation of when it is appropriate to prealert patients with suspected stroke led to frustration between service providers.

### Study strengths and limitations

The present study recruited prospectively but collected data retrospectively and included a sample of real-world patients with stroke attending emergency care in two large urban hospitals. We used a mixed methods approach, which provided greater insight into the mechanisms of prealerting than could have been achieved using quantitative or qualitative methods alone. Individual consent was required to allow data linkage of patient records from different sources which is not otherwise possible in the UK. Of those who consented to participate, data from 100% of secondary care records and 99% of related EMS records were linked. This allowed the association between initial prehospital patient presentation, prealert eligibility and EMS prealerting behaviour to be examined, something which was not possible in previous studies.^8^ ^9^ An attempt was made to sample all patients with suspected stroke but recruitment was limited by the practicalities of engaging with people presenting 24 h a day, 7 days a week. In total, just over half of the potentially eligible patients were recruited and thus the results of this study should be interpreted with caution. The study sample was representative of those in the local stroke population (table 1) and although it was not possible to collect clinical data from non-recruited patients, we see no reason to believe that prealerting would be different in those not recruited to the study. Patients were recruited from urban hospitals and thus the experiences of those attending hospitals in a rural setting, where journey times may be longer, are likely to be different.^17^ The local prealert protocol in place was reflective of current national practice in describing the assessments to carry out in patients with suspected stroke, although national guidelines do not specify definitive prealert criteria.^18^ The findings of this study are therefore only likely to be relevant in healthcare settings which adopt a similar approach.^19^

The accuracy of the quantitative data collected in the present study was reliant on the accuracy with which it was documented. It was not possible to account for scenarios where assessments were conducted but not documented or where information about the patients was only communicated verbally between healthcare professionals. To this end, our multivariate analyses examined a number of factors (supported by the literature^8^ ^9^ and expert opinion) but it was not possible to study factors not recorded in the medical notes, or not prespecified for data collection, which may have influenced the decision to prealert for non-stroke related reasons such as haemodynamic instability, refractory convulsions, injuries sustained as a result of collapse or other adverse events. One prealert eligibility criterion which was included in these analyses as a proxy measure of consciousness, GCS >13, is no longer part of prealert criteria as many patients with dysphasia have a GCS below 13.

It is also important to note that the qualitative data, while adding important context to the quantitative findings, were drawn from a small number of paramedics, most of whom were interviewed in pairs; a larger interview study would have been able to further unpick the complexities of prealerting decisions.

### Study findings in the context of existing literature

We are aware of two previous studies that have examined factors predicting whether or not a prealert message is sent in acute stroke.^8^ ^9^ One of these, carried out by Lin *et al*^9^ in the USA, showed in 90 155 patients that increasing age, Black or Asian ethnicity, history of peripheral vascular disease, increasing stroke severity, travel to a ‘non-academic hospital’ and region of the country were significant predictors of hospital prealerting by EMS staff. Similarly, McKinney *et al*^8^ found that increasing stroke severity, the presence of atrial fibrillation and a positive (vs negative or not performed) Cincinnati Stroke Score^20^ (equivalent to the FAST test)^11^ were all independent predictors of hospital prealerting. The present study was similar in showing that FAST-positive patients, and in particular, those with an increasing number of FAST symptoms (a proxy marker for increasing stroke severity) were associated with increased likelihood of hospital prealerting in acute stroke. The influence of stroke recognition tools on prealerting should be considered in the context of a recent systematic review,^21^ which showed that most prehospital stroke scales (including the FAST test) have limited ability to detect true stroke in routine clinical practice. EMS staff should therefore be cautious about over-reliance on such tools to determine whether a prealert message is sent, without reference to the other (potentially useful) criteria set out in prealert protocols.

Our analysis found that those patients who were seen by a paramedic within 5 h were more likely to be prealerted. Timely presentation and transfer to hospital is a requirement for patients to be eligible for thrombolysis treatment in hospital and therefore it is not unexpected that this predicted the likelihood of hospital prealert. Those patients with a GCS of <13 were also more likely to be prealerted, which is perhaps surprising since it contradicts the localised eligibility criteria for prealerting in acute stroke. This could be explained by the fact that a GCS <13 may represent an indication for prealerting in other conditions, where acute stroke is not suspected.

Although eligibility was not a significant independent predictor of prealerting in our analysis, it is possible that our study was underpowered to detect such an association. We identified 17 patients within our cohort who were eligible, but who were not prealerted. It was difficult to establish from this relatively small sample why such patients were not prealerted, and this was not something which was explored during our qualitative interviews.

### Implications for clinical practice

The present study has shown that in nearly a third of cases studied, patients were prealerted despite not fulfilling the criteria for eligibility set out in localised prealert protocols. It appears that the prealert was given an additional meaning by EMS staff (ie, as a signal of urgency) not envisaged by the original protocol which was not always shared by ED staff, hence leading to disagreements with regard to the appropriate course of action at the point of handover. The handover between EMS and ED staff is important in all emergency situations^22^ but has frequently been found to be inadequate due to poor communication^23^ and a lack of shared understanding between EMS and ED staff.^24^ Perhaps for this reason, EMS staff in the present study spoke favourably about the idea of bypassing the ED and meeting the specialist stroke teams directly. This has been shown to reduce door-to-needle times when effectively implemented in routine clinical practice,^25^ and thus the tendency for EMS staff to prealert even in cases of uncertainty is perhaps understandable.

One solution might be to relax or simplify the criteria for prealerting, aligning the expectations of EMS and ED staff to avoid disagreements upon arrival in hospital. This might be achieved by the introduction of nationally recognised, definitive, prealert criteria to facilitate shared understanding between EMS and ED staff caring for patients with acute stroke. Multidisciplinary education should also be considered to assist this process.^23^ However, prealert protocols are put in place to ensure that hospital resources are used effectively and a high number of inappropriate prealerts could have a negative impact on those patients who are genuinely eligible for thrombolysis if services are stretched beyond capacity. Therefore, identifying alternative ways for EMS staff to convey urgency in this situation may be required if the prealert is to be used strictly within the protocol.

## Conclusions

The present study has found that up to half of the recruited patients presenting with suspected stroke were prealerted by EMS staff, in some cases, against the instruction of locally agreed rapid transfer protocols. Where prealert protocols were not followed, EMS staff reported disagreements with ED staff with regard to the appropriate course of action at the point of handover. Aligning the expectations of EMS and ED staff, perhaps through simplified prealert protocols, could be considered to facilitate more appropriate use of hospital prealerts in acute stroke**.**
