# Supplementary material for: Prevalence and predictors of hospital prealerting in acute stroke: a mixed methods study
Source: Emerg Med J. 2016 Feb 23;33(7):482–8. doi: 10.1136/emermed-2014-204392 (PMC4941194; doi:10.1136/emermed-2014-204392)

Figure s1. Patient pathway for acute stroke in UK hospitals offering a stroke service 24 hours a day, 7 days a week

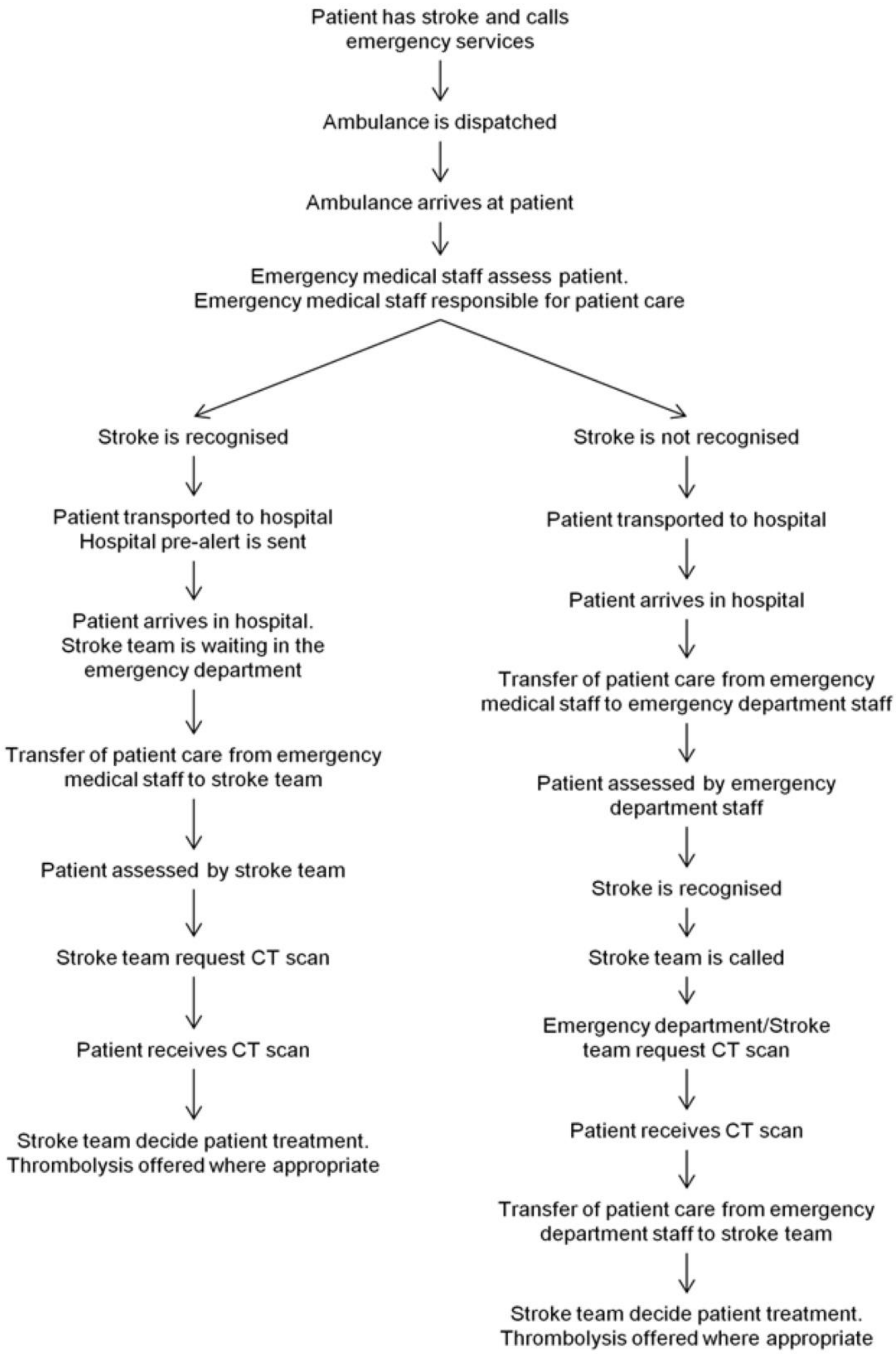

Supplement: Supplementary figure 1 — Patient pathway for acute stroke in UK hospitals offering a stroke service 24 hours a day, 7 days a week [file emermed-2014-204392supp_figure1.pdf]
