# Supplementary material for: Prevalence and predictors of hospital prealerting in acute stroke: a mixed methods study
Source: Emerg Med J. 2016 Feb 23;33(7):482–8. doi: 10.1136/emermed-2014-204392 (PMC4941194; doi:10.1136/emermed-2014-204392)

Figure s2. Local hospital pre-alert criteria

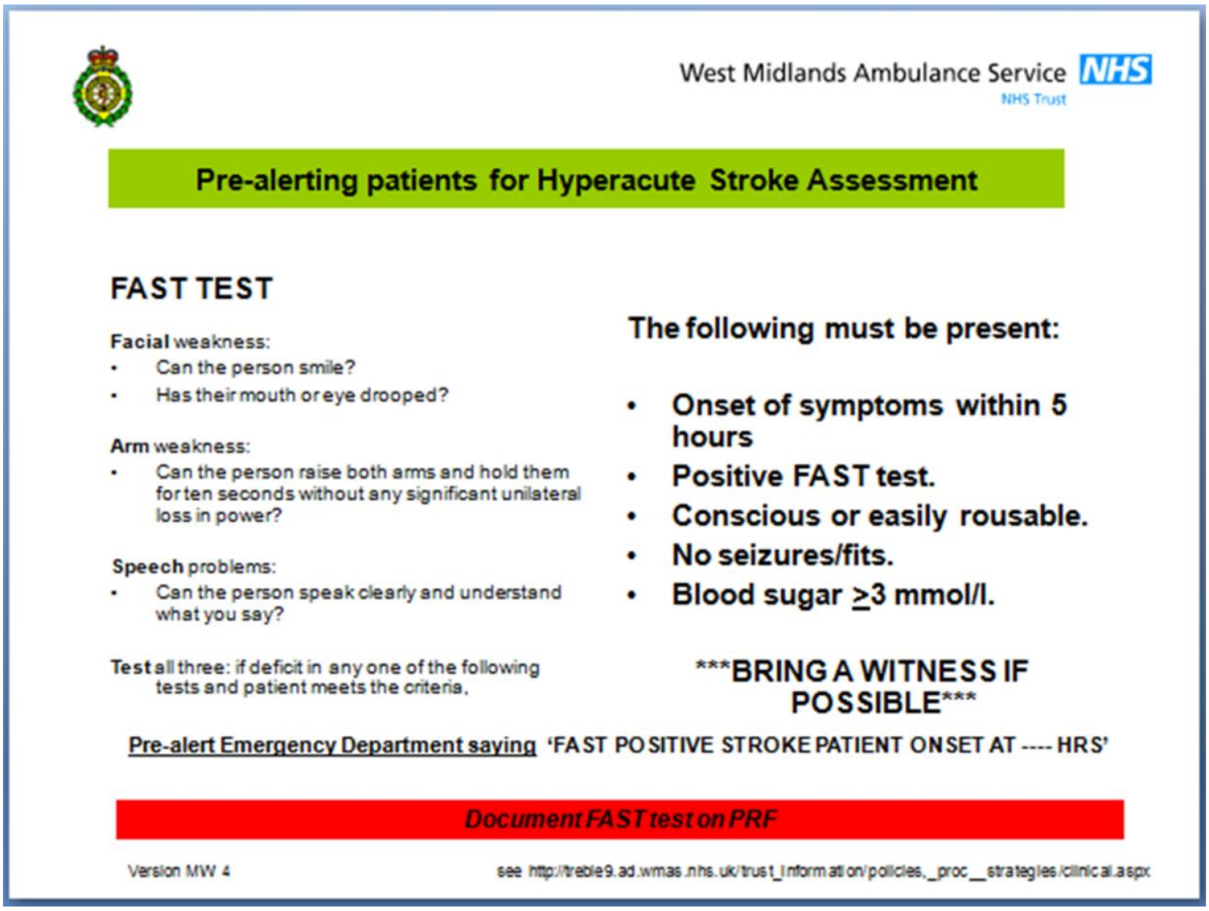

Supplement: Supplementary figure 2 — Local hospital pre-alert criteria [file emermed-2014-204392supp_figure2.pdf]
